# Supplementary material for: Osteoblast Biocompatibility and Antibacterial Effects Using 2-Methacryloyloxyethyl Phosphocholine-Grafted Stainless-Steel Composite for Implant Applications
Source: Nanomaterials (Basel). 2019 Jun 28;9(7):939. doi: 10.3390/nano9070939 (PMC6669514; doi:10.3390/nano9070939)
Supplement: Supplementary file 1 [file nanomaterials-09-00939-s001.pdf]

Supplementary Materials

Osteoblast biocompatibility and antibacterial effects using 2-methacryloyloxyethyl phosphocholine grafted stainless steel composite for implant applications

Dave W. Chen[1,2], Hsin-Hsin Yu[3], Li-Jyuan Luo[4], Selvaraj Rajesh Kumar[3], Chien-Hao Chen[1,2], Tung-Yi Lin[1,2], Jui-Yang Lai[4#], and Shingjiang Jessie Lue[3,5,6,7,*]

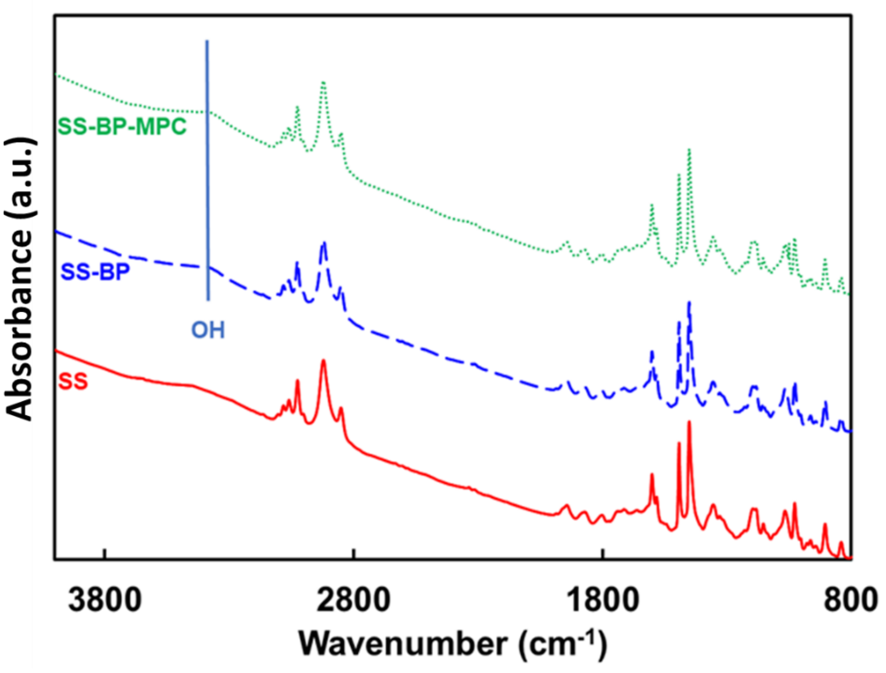

Figure S1: Fourier transform infrared spectra for pristine SS, SS-BP and SS-BP-MPC composites.
